# Preferential Macrophage Recruitment and Polarization in LPS-Induced Animal Model for COPD: Noninvasive Tracking Using MRI

**DOI:** 10.1371/journal.pone.0090829

**Published:** 2014-03-05

**Authors:** Achraf Al Faraj, Asma Sultana Shaik, Mary Angeline Pureza, Mohammad Alnafea, Rabih Halwani

**Affiliations:** 1 College of Applied Medical Sciences, Department of Radiological Sciences, Molecular and Cellular Imaging Lab, King Saud University, Riyadh, Saudi Arabia; 2 Asthma Research Chair and Prince Naif Center for Immunology Research, College of Medicine, King Saud University, Riyadh, Saudi Arabia; University of Giessen Lung Center, Germany

## Abstract

Noninvasive imaging of macrophages activity has raised increasing interest for diagnosis of chronic obstructive respiratory diseases (COPD), which make them attractive vehicles to deliver contrast agents for diagnostic or drugs for therapeutic purposes. This study was designed to monitor and evaluate the migration of differently polarized M1 and M2 iron labeled macrophage subsets to the lung of a LPS-induced COPD animal model and to assess their polarization state once they have reached the inflammatory sites in the lung after intravenous injection. *Ex vivo* polarized bone marrow derived M1 or M2 macrophages were first efficiently and safely labeled with amine-modified PEGylated dextran-coated SPIO nanoparticles and without altering their polarization profile. Their biodistribution in abdominal organs and their homing to the site of inflammation in the lung was tracked for the first time using a free-breathing non-invasive MR imaging protocol on a 4.7T magnet after their intravenous administration. This imaging protocol was optimized to allow both detection of iron labeled macrophages and visualization of inflammation in the lung. M1 and M2 macrophages were successfully detected in the lung starting from 2 hours post injection with no variation in their migration profile. Quantification of cytokines release, analysis of surface membrane expression using flow cytometry and immunohistochemistry investigations confirmed the successful recruitment of injected iron labeled macrophages in the lung of COPD mice and revealed that even with a continuum switch in the polarization profile of M1 and M2 macrophages during the time course of inflammation a balanced number of macrophage subsets predominate.

## Introduction

Chronic Obstructive Pulmonary Disease (COPD) is one of the leading causes of morbidity and mortality worldwide, and represents a substantial economic burden on global health [1]. Alveolar macrophages (AM) play a critical role in the pathophysiology of COPD and are a major target for future anti-inflammatory therapy [2]. They are tissue-resident or recruited cells endowed with high functional plasticity and form the first line of defense against invasive pathogens. This allows them to acquire either pro- or anti-inflammatory as well as tissue remodeling phenotypes during the course of inflammation. The environmental conditions, degree and type of the inflammatory process will essentially drive their polarization to either M1 or M2 macrophages to consequently terminate and resolve the inflammation [3]. Considerable efforts have been made in the last years to better understand the heterogeneity of macrophages, their diverse role in lung inflammation and tissue remodeling and the molecular mechanisms that regulate macrophage polarization and plasticity under both *in vitro* and *in vivo* conditions [4]–[6].

While it becomes evident that the macrophage activation states are plastic in that cells can switch back and forth between them in a phenotypic continuum, depending on the environmental milieu, the exact mechanism that governs this polarization and a net characterization of each subset *in vivo* still needs further investigation. The visualization of macrophage migration could be of high relevance for both diagnostic purposes and evaluation of therapeutic interventions. In addition, their ability to be recruited in the sites of inflammation, infection, and tissue degeneration makes them fascinating vehicles to deliver contrast agents as diagnostic purposes or drugs as therapeutic modalities [7].

On the other hand, difficulties in monitoring the pathogenic processes involved in the progression of lung disease *in vivo* have hampered the design and study of therapeutic interventions. In order to improve the identification and long-term care for patients with COPD, new techniques are required for better assessment of morphological and functional impairment and contribution of each aspect to the patients’ symptoms. Therefore, a noninvasive imaging modality that enables *in vivo* detection of a single inflammatory cell population to understand the physiological process in COPD is highly required for both diagnostic purposes and therapeutic applications. Magnetic resonance imaging (MRI) is well suited for this purpose as it allows both whole body examination and subsequent detailed depiction of host organs with excellent anatomic resolution and soft-tissue contrast. Recently, macrophage imaging using MRI coupled with the use of super-paramagnetic iron oxide (SPIO) nanoparticle as intracellular contrast agent has emerged as a promising noninvasive technique for pre-clinical and clinical studies of several inflammatory diseases [8]–[10]. The strong magnetic susceptibility effect (T2- and T2*-weighted contrast) of SPIO nanoparticles can provide the labeled cells with sufficient magnetization to become sensitively detectable by MRI. SPIO nanoparticles have equally shown no adverse cytotoxicity effect to the cells and they do not affect their proliferation, differentiation or other functions [11], [12] and consequently some iron oxide nanoparticles have been approved for clinical applications [13].

Noninvasive tracking of macrophage in pulmonary inflammatory diseases were limited using MRI modality because of the difficulties to image this organ due to factors such as signal loss due to cardiac pulsation and respiration, susceptibility artifacts caused by multiple air-tissue interfaces and low proton density. However, with ongoing technical improvements in gradients systems as well as the development of innovative pulse sequence techniques such as ultra-short echo time (UTE) sequence [14], macrophage detection using MRI may open novel perspectives for both imaging, diagnostic and therapeutic interventions for chronic respiratory diseases such as COPD. Recently, new MR imaging techniques using either injection of perfluorocarbons in ^19^F MRI [15] or the T1 effect of injected very small SPIO nanoparticles using UTE sequence [16] were reported for detecting macrophage infiltration in models of pulmonary diseases. The purpose of our study was to monitor and evaluate the migration of intravenously injected, differently polarized, M1 and M2 iron labeled bone marrow derived macrophage subsets to the lung of a COPD animal model. In addition, the polarization state of macrophages subpopulations was assessed once they have reached the inflammatory sites in the lung.

## Materials and Methods

### Animals and COPD Model

Female Balb/c mice (20–22 g) were obtained from the University’s main animal care center. All experiments were performed in accordance with the National guidelines for the care of laboratory animals and the study was approved by the Ethical Committee of the College of Applied Medical Sciences (agreement number: CAMS05/3334). Animals were divided into eight groups (G): Four of them (COPD groups) have received an intrapulmonary instillation (0.5 mg.kg^−1^; V = 100 µl) of LPS from E. coli (Santa Cruz Biotechnology, Inc., CA, USA) using a MicroSprayer aerosolizer (Penn-Century Inc., PA, USA). The other four groups (Ctrl groups) have received an intrapulmonary instillation of physiological saline solution (V = 100 µl). At 48 h after LPS challenge or saline pulmonary exposure, mice were intravenously injected with either saline, SPIO ([Fe] = 4 mM corresponding to 16 mmol of iron kg^−1^; V = 100 µl), or iron labeled *ex vivo* polarized M1 or M2 macrophages (1×10^6^ cells of each subpopulation were suspended in 100 µl phosphate buffered saline (PBS) solution). During the different experimental procedures, each animal was anesthetized by intramuscular administration of a mixture of 0.1 mL of 4 mL of ketamine (500 mg/mL), 1 mL of xylazine (2%), and 5 mL of physiological serum.

### Macrophages Polarization

Bone marrow (BM) derived M1 and M2 macrophages (BMDM) were obtained as previously reported [17]. Briefly, BM cells from tibiae and femora of donor mice were incubated for 7 days at 37°C in complete IMDM medium supplemented with 10 ng/ml of macrophage clone stimulating factor (R&D systems, Abingdon, UK) to obtain adherent nonpolarized-M0 macrophages. Macrophage polarization was then induced by incubating adherent M0 cells for 20 h at 37°C in complete IMDM medium supplemented with 1 ng.ml^−1^ LPS (Santa Cruz Biotechnology, Inc., CA, USA) and 10 ng.ml^−1^ INFγ (R&D systems, Abingdon, UK) to obtain M1-polarized cells or with 10 ng.ml^−1^ IL-10 and 20 ng.ml^−1^ IL-4 (R&D systems, Abingdon, UK) to obtain M2-polarized macrophages.

### SPIO Nanoparticles Characterization

Dextran coated iron oxide nanoparticles (Nanomag-D-spio, Micromod Partikeltechnologie GmbH, Germany) functionalized by the addition of polyethylene glycol (PEG) with amine terminal (NH_2_) were preferred for efficient macrophages labeling [18], [19]. We have previously reported that these nanoparticles showed an enhanced labeling efficiency of macrophages with a better biocompatibility [20]. These nanoparticles have a dextran coating of 40,000 g/mol and a PEG length of 300 g/mol for a total diameter of 100 nm with an iron oxide crystallite diameter of 10–13 nm according to the supplier specifications. Iron concentration and nanoparticles size were re-checked as quality control step before use (data not shown). The electrical surface charge of the nanoparticles was determined by measuring the zeta potential in ultrapure water at 25°C using a Zetasizer Nano ZS90 (Malvern Instruments, Malvern, UK) and results were expressed as zeta potential (mV) ± SD on average of three measurements. The longitudinal (r1) and transverse (r2 and r2*) relaxivities of the used magnetic nanoparticles were then measured at 25°C (File S1) on tubes containing a suspension of nanoparticles at different iron concentrations (0, 0.10, 0.15, 0.20, 0.40, 0.60, 0.80 mM) using a 4.7T Pharmascan 47/16 Bruker magnet interfaced to ParaVision 5.1 software (Bruker Biospin GmbH, Rheinstetten, Germany).

### Macrophages Labeling and Uptake Quantification

M1 or M2 bone marrow derived macrophages were labeled with SPIO nanoparticles at an extracellular iron concentration of 2 mM with 1 h incubation time at 37°C, which has been chosen as the best compromise between labeling efficiency and biocompatibility to the cells. Macrophages were incubated in serum-free RPMI medium (Gibco, Lifetechnologies, CA, USA) to avoid proteins interactions with the uptake mechanism [21]. The incubation step was followed by an overnight chase period in SPIO-free culture medium to allow sufficient time for iron oxide internalization. Labeling efficiency was quantified using Ferrozine-based spectrophotometric assay that allowed accurate determination of iron content in labeled cells by comparing to a standard calibration curve. Briefly, 2×10^5^ labeled cells were digested with hydrochloric acid and absorbance was measured at 351 nm using Multiskan Go Spectrophotometer (Thermo Scientific, NH, USA). Using a calibration curve of the absorbance vs. iron concentration of the SPIO nanoparticles (Figure S1) prepared under the same experimental protocol, the iron concentration in the labeled macrophages was accurately determined and expressed as pg of iron per cell.

### *In vitro* Cytotoxicity Evaluation

#### Cell proliferation and viability

Cell proliferation and viability was evaluated using a cell proliferation assay kit (Merck Millipore, MA, USA) according to the manufacturer protocol. Briefly, SPIO labeled M1 and M2 macrophages were placed in triplicates in a 96-well plate at a concentration of 10^4^ cells/well. Following the overnight chase period, absorbance was measured using Multiskan Go Microplate Spectrophotometer (Thermo Scientific, NH, USA) with a test wavelength of 440 nm and a reference wavelength of 630 nm. The relative percentage of cell viability for each condition was calculated related to unlabeled M1 and M2 macrophages.

#### Reactive Oxygen Species (ROS) generation

The release of ROS in SPIO labeled M1 and M2 macrophages subsets were detected using 2′,7′ dichlorofluorescein diacetate (DCFDA), a fluorogenic dye that measures hydroxyl, peroxyl and other ROS activity within the cell. After diffusion into the cell, DCFDA is deacetylated by cellular esterases to a non-fluorescent compound, which is later oxidized by ROS into 2′, 7′ dichlorofluorescin (DCF), a highly fluorescent compound. Briefly, SPIO labeled M1 and M2 macrophages were seeded in triplicates in black 96-well plate at a concentration of 10^4^ cells/well. Following the overnight chase period, 100 µl of 25 µM DCFDA was incubated at 37°C for 15–30 min and fluorescence (excitation 485 nm, emission 535 nm) were measured using a Synergy 2 Multi-Mode Microplate Reader (BioTek, VT, USA) to determine the change as percentage from control after background subtraction.

#### iNOS and arginase1 activity determination

As markers of M1 and M2 macrophages respectively, the levels of iNOS and Arginase1 activity were determined before and after labeling with iron oxide nanoparticles. iNOS activity was measured on the basis of nitrite accumulation in macrophages supernatants, and Arginase1 activity was determined by measuring arginine derived urea in macrophages extracts as previously described [22]. Absorbance was measured at 540 nm using a Multiskan Go microplate reader (Thermo Scientific, NH, USA) and the concentration of nitrite and urea in the samples was determined by comparison with a standard curve of sodium nitrite (1 to 200 µM) and urea (1 to 100 µg/mL) respectively.

### Macrophages Noninvasive Monitoring using MRI

To validate the noninvasive detection and assess the biodistribution of intravenously injected iron labeled M1 or M2 macrophages in control and COPD animal model, MRI investigation was performed on the 4.7T magnet.

#### *In vivo* biodistribution in abdominal organs

M1 and M2 macrophage biodistribution (1×10^6^ cells) was monitored using a gradient echo sequence (TR/TE = 300/3 ms, flip angle = 30°, 4 averages, total acquisition time = 5 min) with a pixel resolution of 100×100 µm and a 1 mm axial slices positioned over organs of interest such as the liver, spleen and kidneys. Macrophages biodistribution was compared to free SPIO nanoparticles administrated at a dose of 16 mmol.kg^−1^, which approximately correspond to the quantity of iron carried by the injected macrophages. Abdominal MR Imaging protocol was repeated at −1 h prior- and at 5 min, 2 h, 24 h, 48 h, 72 h and 7 days post- injection of labeled macrophages or free iron oxide nanoparticles.
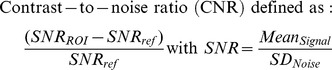
where ref denotes an external water tube reference and SD is the standard deviation of the noise in the image, was measured in the different organs of interest. Within the liver, regions of interest (ROI) were drawn around apparent vascular structures and these regions were subtracted from the map to retain liver parenchyma only. ROIs encompassing the whole spleen or the two kidneys were manually selected for signal measurement.

#### Macrophages detection in the lung

To track macrophages homing to the inflammatory sites in the lung, a free-breathing imaging protocol using a radial UTE sequence (TR/TE = 100/0.4 ms, flip angle = 15°, 4 averages, total acquisition time = 5 min 21 s) with 100×100 µm pixel resolution and 1 mm axial slice thickness was optimized to allow both detection of iron labeled macrophages and visualization of inflammation progression. Pulmonary MR Imaging protocol was repeated at −48 h prior- (i.e., before LPS induction), −10 min (i.e., before injection of labeled macrophages or free iron nanoparticles) and at 10 min, 2 h, 24 h, 48 h, 72 h and 7 days post- injection. SNR was measured in ROI drawn around apparent vascular structures and these regions were subtracted from the map to retain lung parenchyma only. Slices were positioned at the same position for all animals during the 1-week follow-up study.

### Characterization of Macrophages Polarization States

Mice from the various treatment groups were sacrificed at 2 h post macrophages injection by lethal overdose injection of anesthesia. A combination of cytokines and chemokines secretion measurement using ELISA, flow cytometry analysis, and immunohistochemistry investigations were performed in the lung to assess the macrophages polarization once they have reached the inflammatory sites.

#### Cytokines and chemokines secretion measurement

Lungs (n = 5 in each group) were cannulated via the trachea and lavaged three times with 1 ml PBS containing 0.5 mM EDTA. Broncho-alveolar lavage (BAL) supernatant was then collected and the levels of IL-4, IL-12, CXCL-10, CCL-22 were quantified by an enzyme-linked immunospecific (ELISA) assay following the manufacturer’s protocol (R&D systems, Abingdon, UK).

#### Macrophages surface membrane expression

Macrophages were obtained from mouse lungs after sacrifice and prepared for flow cytometry as per the protocol described elsewhere [23]. Briefly, the dissected lungs were held in complete RPMI medium supplemented with 25 mM HEPES. Lungs were minced in complete medium and the filtrates were centrifuged (1500 rpm for 5 min). The number of cells in the supernatant were determined and then the solutions passed through magnetic activated cell sorter (MACS) microbeads and through MACS LD or MS columns, as per the manufacturer’s protocol (Miltenyi Biotec, Germany). The eluted unbound and bound cell fractions were first separated, and after a repeat cell count, they were tested for different markers by flow cytometry. The cells were then washed, fixed and permeabilized with Cytofix/Cytoperm (BD Biosciences, NJ, USA), stained with conjugated anti-mouse antibodies as follows: Alexa Fluor 488-labeled anti-CD86, Alexa Fluor 647-labeled anti-CD197, RPE-labeled anti-CD23, and FITC-labeled anti-CD206 (AbD Serotec, UK), and analyzed with BD LSR II flow cytometer using DIVA Software. Data were collected (10,000 events each time) with a FACSCalibur flow cytometry system and analyzed with the CellQuest Software (BD Biosciences, NJ, USA).

#### Lung immunohistochemistry and prussian blue staining

Lungs (n = 3 in each group) were removed and fixed overnight in 4% paraformaldehyde. They were then embedded in paraffin for histological analysis and sets of four consecutive 5 µm thick sections were obtained. After preparation of tissue sections, immunohistochemistry was performed using the following primary antibodies: F4/80 rat monoclonal IgG (1∶100), Arginase goat polyclonal antibody (1∶100) and NOS2 rabbit polyclonal antibody (1∶1000) (Santa Cruz Biotechnology, Inc., CA, USA). Respective mouse ABC staining systems were used as sources for secondary antibodies and as per the protocol specified by the manufacturer. Briefly, tissue sections were deparaffinised and incubated with primary antibodies diluted in blocking sera. After incubations, slides were washed and incubated with biotinylated secondary antibodies followed by enzyme reaction with the addition of peroxidase substrate. The sections were counterstained with haematoxylin, dehydrated in a series of alcohol and xylene solutions, mounted and observed under a BX53 Olympus microscope.

To check for the presence of iron oxide nanoparticles and their co-localization with macrophages, a Prussian blue (PB) staining protocol was followed. Sections were deparaffinized, hydrated, and then stained in a 1∶1 solution of 20% aqueous solution of hydrochloric acid and 10% aqueous solution of potassium ferrocyanide. Following washing of slides, the sections were counterstained with neutral red, dehydrated with a series of alcohols, cleared in xylene and observed after mounting.

### Statistical Analysis

Data presented as the mean standard deviation were analyzed by t-test using SPSS software (SPSS, IL, USA). For MRI data, nonparametric statistical tests: Kruskal–Wallis for unpaired groups and Friedman test for comparison between different time points were used. A p-value <0.05 was considered significant for all tests.

## Results

### Efficient Labeling of M1 and M2 Polarized Macrophages

**Figure 1:**
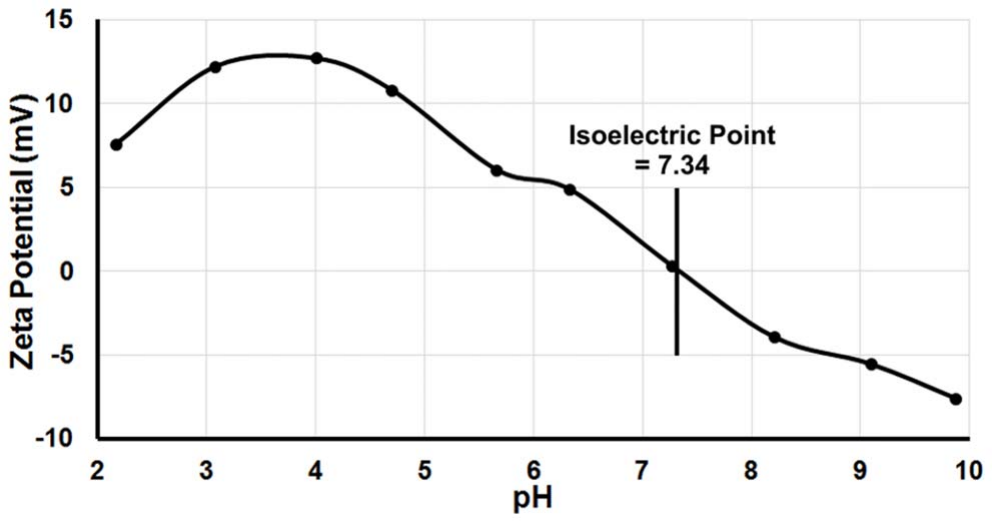
Zeta potential of amine-modified PEGylated dextran-coated iron oxide nanoparticles assessed in ultrapure water at 25°C.

Zeta potential analysis of the iron oxide nanoparticles revealed a slightly positive value (1.65±0.5 mV) at neutral pH with an isoelectric point at 7.34 (Figure 1). Measurement of r1, r2 and r2* relaxivities values at 4.7T exhibited a high r2 and r2* values (229.48±3.44 and 264.52±4.16 mM-1.s-1) and a negligible r1 value (0.06±0.01 mM-1.s-1). SPIO nanoparticles labeling of M1 and M2 polarized macrophages with an extracellular iron concentration of 2 mM and 1 h incubation time showed an uptake of 16.41±1.07 and 20.48±1.2 pg/cell for M1 and M2 macrophages, respectively.

### *In vitro* Cytotoxicity Evaluation and Macrophages Polarization

**Figure 2:**
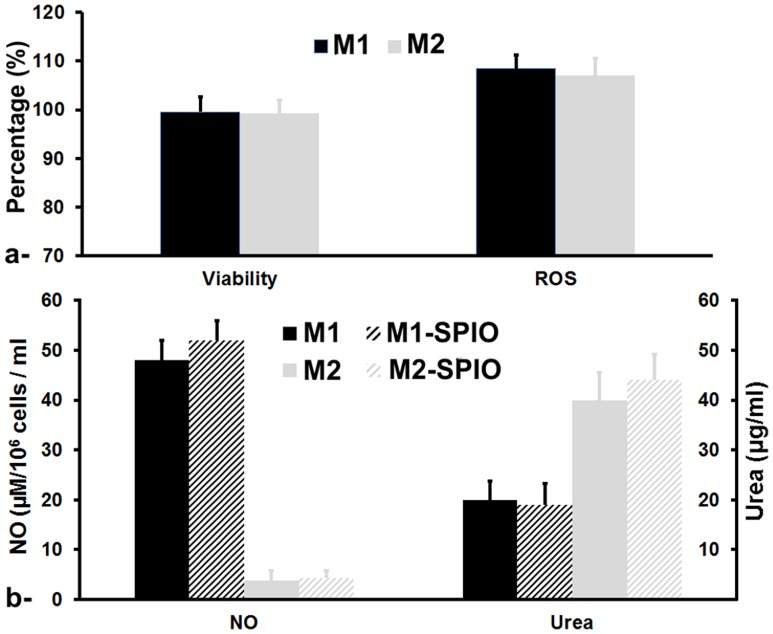
Relative percentage of viability and reactive oxygen species generation of M1 and M2 SPIO labeled macrophages compared to unlabeled macrophages subsets after the overnight chase period (a). Nitric oxide (NO) release as marker of iNOS activity (left) in M1 macrophages and Arginine-derived urea production (right) as marker of Arginase1 activity in M2 macrophages (b). Error bars are standard deviation of triplicates.

The relative viability percentage showed a 99.58±3.1% and 99.19±2.8% viability for M1 and M2 macrophages respectively. Approximately, 7 to 8% increase of ROS generation was observed for both M1 and M2 labeled macrophages (108.42±2.9% and 107.03±3.5%, respectively) comparing to non-labeled subsets (Figure 2a).

No statistically significant variation in the levels of NO and urea was observed after SPIO labeling under the used experimental conditions. As expected, a high level of NO was perceived for M1 macrophages with negligible level for M2 subsets (Figure 2b). Conversely, a 2-fold level of urea was observed for M2 macrophages compared to M1 subsets.

### Noninvasive Tracking of Macrophages Subsets after i.v. Administration in COPD Animal Model

**Figure 3:**
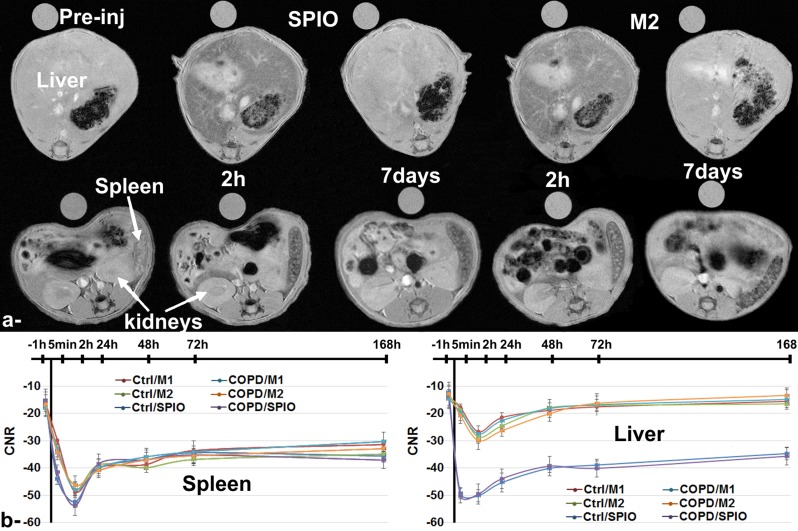
MR images acquired using susceptibility-weighted gradient echo sequence showing the liver (upper row) and the spleen and kidneys (lower row) pre-injection (−1 h) and at 2 hours and 7 days post-injection of either free SPIO or SPIO labeled M2 macrophages in control mice groups (a). Contrast-to-noise (CNR) variation during the 7 days follow-up study for the spleen (left side) and the liver (right side) before and after intravenous injection of either free SPIO, SPIO labeled M1 or M2 macrophages in control and COPD animal groups (b). Error bars are standard deviation of triplicates.

Using susceptibility-weighted gradient echo sequence, M1 and M2 macrophages were mainly detected within 2 hours post-injection in the spleen with 48.7±3.6% and 47.9±2.4% CNR attenuation and to a lesser extent in the liver with 26.7±3.1% and 28.6±2.7% in M1 and M2 macrophages injected controls groups, respectively (Figure 3). No statistically significant difference was observed between control and COPD groups. Conversely, in both control and COPD groups, free SPIO nanoparticles were readily detected within 5 minutes post-injection at a similar level in both the spleen and liver with 52.3±2.2% and 50.1±3.5% CNR attenuation in control groups respectively.

CNR attenuation progressively started decreasing for the different groups 24 hours post-injection in both organs, however, it was still statistically significant after the 1-week follow-up investigations in both spleen and liver for free SPIO injected groups and in just the spleen for macrophages injected groups. Finally, no statistical changes were detected in kidneys in all the different groups (data not shown).

**Figure 4:**
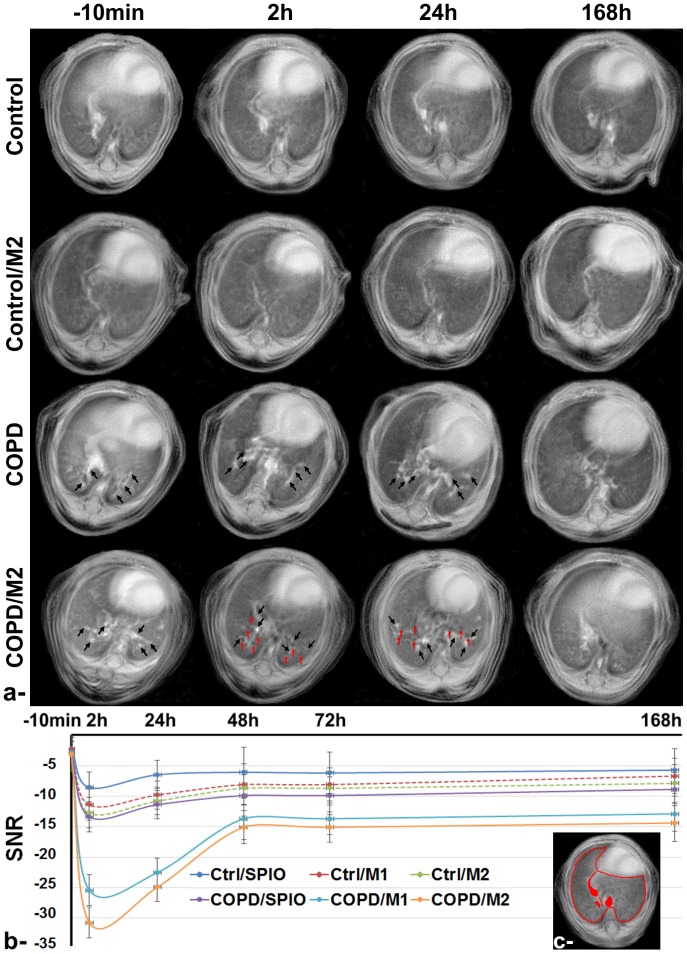
MR Images acquired using ultra-short echo time (UTE) sequence of control and LPS-induced COPD lungs, with or without injection of M2 macrophages (a). From top to bottom: Control, Control/M2, COPD, COPD/M2 groups imaged at (from left to right) −10 min, 2 h, 24 h and 168 h post M2 macrophages injection. Black arrows highlight the inflammatory regions in COPD groups and red arrows highlight the presence of void signal dots related to higher macrophage infiltrations in the inflammatory lungs. Signal-to-noise (SNR) attenuation of lung parenchyma, during the 7 days follow-up study, measured before and after intravenous injection of either free SPIO, SPIO labeled M1 or M2 macrophages in control and COPD mice (b). Error bars are standard deviation of triplicates. Representative regions of interest (ROI) which were drawn around apparent vascular structures (filled in red) and subtracted from the map to retain lung parenchyma (c).

On the other hand, pulmonary inflammation induced by the intrapulmonary instillation of LPS 48 hours prior to macrophages injection was successfully detected in the lung of COPD groups using UTE sequence as hyper-intensity regions homogenously distributed in the different lobes of the lung (black arrows, Figure 4). Remarkably, M1 and to a higher extent M2 labeled macrophages were detected in the lung with the most prominent effect readily observed within 2 hours post injection with a SNR attenuation of 25.4±2.5% and 30.7±2.8% for COPD/M1 and COPD/M2 groups, respectively. Visually, higher macrophages infiltrations were localized mainly near the inflammatory sites in the lung (red arrows, Figure 4). The attenuation of signal in both M1 and M2 COPD groups gradually decrease with time to become non-statistically significant from control groups at 48 h post-injection investigation time point. Conversely, no statistical variation was observed for free-SPIO injected control and COPD groups and for M1 and M2 macrophages injected control groups over the 1 week follow-up.

### Levels of Cytokines in BAL Samples

**Figure 5:**
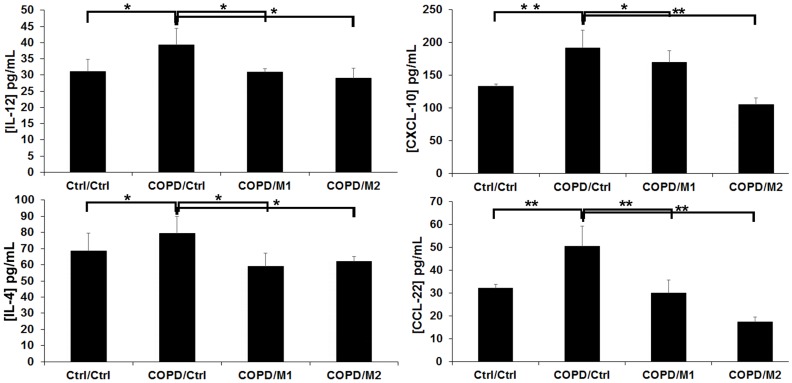
Interleukins (IL-12, IL-4) and Chemokines (CCL-22 and CXCL-10) levels quantified by ELISA assay obtained from BAL samples of ctrl/ctrl, COPD/ctrl, COPD/M1 and COPD/M2 animal groups at 2 h post macrophage injection. Data expressed as mean ± SD, n = 5 per group. *p<0.05; **p<0.01.

Quantification of Interleukins (IL-12 and IL-4) and Chemokines (CCL-22 and CXCL-10) levels in BAL samples revealed that at 48 hours post-LPS exposition to the lung a significant increase in both Interleukins and Chemokines was observed compared to control group (Figure 5). Interestingly, the intravenous administration of M1 and M2 macrophages has been shown to substantially decrease their levels once reached the lung at 2 h post-injection.

To quantify the number of macrophages that have reached the lung and evaluate their polarization profile, iron labeled macrophages were sorted from total cellular extracts of mice lungs 2 hours post injection using magnet attraction (MACS assay). Of the 1×10^6^ injected macrophages, around 0.32×10^6^ and 0.35×10^6^ of retained iron labeled cells were found in the COPD lung at 2 hours post injection of M1 and M2 macrophages respectively.

**Figure 6:**
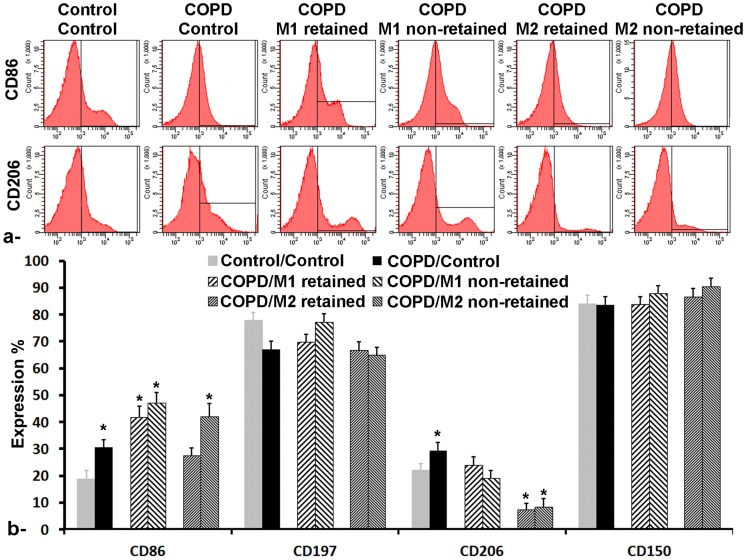
Flow cytometry analysis of alveolar macrophages in control/control, COPD/Control (48 h post LPS intrapulmonary exposition), COPD/M1 and COPD/M2 (2 h post-injection of iron-labeled macrophages) groups for both retained (i.e., iron loaded) and retained fractions. a- representative histogram of CD86 expression (higher row) and CD206 (lower row). b- Surface membrane receptor expression percentage of CD86, CD197, CD206 and CD150. Error bars are standard deviation of triplicates. *p<0.05.

Macrophages were then labeled with CD86, CD197, CD206 or CD150 FACS antibodies and the expression percentages of their surface membrane receptor (i.e., CD86 and CD197 for M1 and CD206 and CD150 for M2) were analyzed using flow cytometry. At 48 hours post LPS exposition to the lung, an over-expression of both CD86 and CD206 (30.7±2.7% and 29.3±3.1% respectively in COPD/Control group compared to 18.8±3.2% and 22.1±2.3% in Control/Control group) was observed (Figure 6a) while no statistical variation was detected for CD197 and CD150 (Figure 6b). Interestingly, following injection of M1 macrophages, the expression of CD86 increased to 41.8±4.1% in M1-retained fraction (i.e., iron loaded) and 47.2±3.7% in M1-non-retained fraction whereas no variation in the expression of the different markers was perceived. Conversely, following injection of M2 macrophages, an over-expression of CD86 was observed in just the M2-non-retained fraction (41.9±4.9%) compared to M2-retained fraction (27.6±2.8%) while a down-expression of CD206 was observed in both M2- retained and non-retained fractions (7.4±2.3% and 8.3±3.1% respectively). Finally, similarly to M1 injected COPD group, no variation was detected for CD197 and CD150 in M2 injected COPD group in both retained and non-retained fractions.

**Figure 7:**
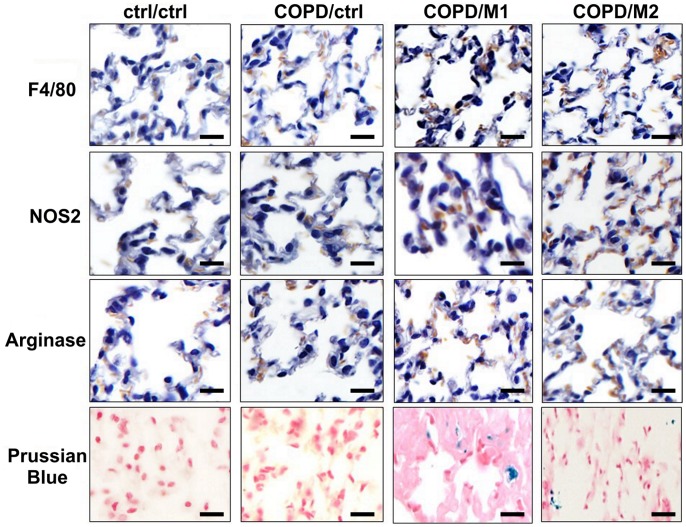
Examples of Immunohistochemical (IHC) staining of lungs sections of ctrl/ctrl, COPD/ctrl, COPD/M1 and COPD/M2 groups at 2 hours post-injection of macrophages using: F4/80 (first row) as universal marker for macrophages, iNOS (second row) and Arginase1 (third row) as marker for M1 and M2 macrophages respectively and of Prussian blue iron staining (fourth row) in adjacent sections revealing the presence of iron oxide nanoparticles (blue dots) in macrophages injected groups. Scale bar: 100 µm.

Immunohistochemistry revealed the successful recruitment of macrophages into the lungs of a COPD animal model (Figure 7, COPD/M1 and COPD/M2 groups as observed by an increase in F4/80 marker) compared to either COPD/ctrl group or to a higher extent ctrl/ctrl group. Identification of M1 and M2 macrophage subsets homing to inflammatory sites in the lungs was assessed by staining for iNOS and Arginase1 markers. Screening of immunohistochemistry slides by three independent observers (more than 5 years of experience) revealed almost equal numbers of M1 macrophages and M2 macrophages at 2 hours post-injection. This was also confirmed through the observations following Perl’s staining of lung sections that revealed co-localization of iron labeled M1 and M2 macrophages (i.e., Prussian blue reaction) within the lungs of injected COPD mice.

## Discussion

This study comprehensively evaluated the biodistribution of differently polarized M1 and M2 iron labeled macrophage subsets in a COPD animal model with the objective of noninvasively detecting their homing to the lung using MRI and assessing their polarization profile after they have reached the sites of inflammation. To demonstrate this, the M1 and M2 polarized macrophages subsets were first successfully differentiated from the bone marrow derived cells of a donor mice. To enable their enhanced detection using noninvasive MRI, macrophages labeling was performed using amine-modified PEGylated dextran-coated iron oxide nanoparticles that were checked for biocompatibility before their *in vivo* administration. It is well known that the size and the surface charge of SPIO nanoparticles govern cellular internalization and their distribution [24], [25]. In line with previous studies [20], [26], the SPIO nanoparticles with an overall size in the range of 100 nm allowed better internalization with optimal biocompatibility in this study. In addition, being positively charged, our nanoparticles showed efficient penetration [27] and enhanced labeling efficiency which might have allowed a higher affinity with the negatively charged cell surface [20].

SPIO nanoparticles used in this study showed a high r2 and r2* values (229.48±3.44 and 264.52±4.16 mM-1.s-1) at 4.7T which were greater than the current clinically approved negative contrast agents with a negligible r1 value [28]. This helped in obtaining a largely sufficient contrast effect for a better detection using MRI. With their high r2/r1 ratio, amine-modified PEGylated dextran-coated iron oxide nanoparticles offered excellent negative contrast effect and better detection of small aggregates or single cells. After validating the efficiency of labeled macrophages, we established that the amine-modified PEGylated dextran-coated iron oxide nanoparticles did not modify the functionality, health and polarization of macrophages subpopulations. Furthermore, ROS generation was also measured as an alternative index for biocompatibility. Results showed that the tested labeled macrophages were biocompatible – the observed low-level ROS generation may help in the induction of various biological effects that are involved in regulating the normal cell functions after external stimuli [29], [30]. In addition, quantification of iNOS and arginase1 activity, as markers of M1 and M2 macrophages respectively, demonstrated successful polarization of bone-marrow derived macrophages and confirm that their magnetic labeling with the used SPIO nanoparticles do not modify their polarization profile. Similar findings were observed in our previous study [17] using flow cytometry analysis where no variation in the expression of surface membrane receptor was detected following iron oxide labeling.

In order to assess the biodistribution and clearance of intravenously injected macrophage subsets in control or COPD mice, MRI investigation was first performed on abdominal organs of interest (i.e., spleen, liver and kidneys) of mice receiving 1 million of magnetically labeled M1 or M2 macrophages. While both macrophage subsets were mainly detected in the spleen and to a lower extent in the liver within 2 hours after their intravenous injection with a similar biodistribution kinetic, a slightly prominent effect was observed for the M2 groups, most probably due to their higher iron uptake, which is in line with their role as debris scavengers [31]. No variation in the migration dynamic of macrophages was observed between control and COPD groups. This suggests that the intrapulmonary instillation of LPS to the lung of Balb/c mice, under the current experimental conditions and investigation time course, has produced a local inflammation in the lung with no systemic effect in abdominal organs [32] even that activation of circulating inflammatory cells and increased circulating levels of inflammatory cytokines may occur [33].

Then, to sensitively detect the homing of macrophage subpopulations to the lung of LPS-induced COPD mice, a radial UTE sequence with a very short echo time (e.g., TE = 0.4 ms) was implemented. This free-breathing imaging protocol has provided a good contrast for the detection of fluid secretion related to inflammation, allowed attaining sufficient MR signal in lung parenchyma and a good contrast for iron labeled macrophages detection in a noninvasive imaging protocol. Having a similar biodistribution profile in the abdominal organs, M1 and M2 macrophages have been also found to home similarly to the sites of inflammation in the lung, although a higher SNR attenuation was observed for M2 macrophages. We believe that this effect could be due to their higher load of SPIO nanoparticles rather than a higher quantity of M2 cells reaching the lung.

Following the experiments that validated for the first time the possibility of tracking and non-invasively monitoring intravenously injected bone marrow derived M1 and M2 macrophages subpopulations to the sites of inflammation in LPS-induced COPD, we further investigated whether one subpopulation of macrophages can preferentially reach the inflammatory sites and/or if there was a switch in their polarization profile. SPIO labeled macrophages were observed as early as 2 hours post injection and primarily located in the lung parenchyma. At that time point, we have quantified the levels of Interleukins (IL-12 and IL-4) and Chemokines (CCL-22 and CXCL-10) levels in BAL samples from ctrl/ctrl, COPD/ctrl, COPD/M1 and COPD/M2 groups. As expected, LPS caused a significant increase in the levels of all cytokines at 48 hours post LPS exposition with a continuum balance in the polarization of macrophages at this stage of inflammation. M1 cytokines (IL-12 and CXCL-10) were produced to allow pro-inflammatory actions while M2 cytokines (IL-4 and CCL-22) were released to start modulating the inflammatory process and aid tissue repair [34]. However, once reached the lung at 2 h post-injection, the intravenous administration of M1 and M2 macrophages has been shown to substantially decrease their levels.

Cytokines production largely affect macrophage phenotypes in the tissue environment. Switching a macrophage from an inflammatory cytokine producing cell to an anti-inflammatory producing cell may hold the key to induced non-immune responses or peripheral tolerance. LPS may have caused M1 macrophages to produce high levels of IL-12 which cause inflammatory T cell responses, in addition, there could have been a shuffling of M2 macrophages to M1 macrophages to promote inflammation [35]. CCL-22, a chemokine produced by macrophages [36] – most commonly M2 subtype has been reported to be increasingly expressed in COPD, with its up-regulation also being enhanced by IL-4 [37], [38]. The increase in CCL-22 during the initial stages of inflammation is in line with the results reported by Ying et al [37] and Frankenberger et al 2011 [39] who showed a similar pattern upon LPS induced inflammation in COPD and this could help in recruitment of effector T-lymphocytes to sites of inflammation [40]. The increase in IL-4 at this stage of inflammation could be due to its potential role in tissue repair that inhibits activation of macrophages to M1 subtype – this may take a predominant role in counteracting inflammation by increasing the number of M2 or repair macrophages.

On the other hand, quantification of the number of intravenously injected magnetically labeled M1 and M2 macrophages using magnet attraction has shown an equal number of cells reaching the lung (MACS retained fraction) at 2 hours post-injection. This result demonstrates for the first time that during LPS-induced COPD model, there could be equal proportions of macrophage subpopulations homing to the site of inflammation in the lung in just 2 hours and this migration can be monitored using an non-invasive imaging modality.

Moreover, flow cytometry analysis has confirmed the presence of both M1 and M2 macrophages in the lung, at this stage of LPS-induced inflammation, with an over-expression of both CD86 (M1 marker) and CD206 (M2 marker). Interestingly, following injection of M1 macrophages, an over-expression of CD86 was detected in not only the retained (i.e., iron labeled M1 macrophages that have reach the lung) but also the non-retained fractions. This finding suggest that the injected SPIO-labeled M1 macrophages might be recognized as foreign bodies and hence stimulated the activation of dendritic cells (DC), which also highly express CD86 marker, and therefore adding to the main population in the non-retained fraction. Consistent with these expectations, the injection of SPIO-labeled M2 macrophages was also found to over-express CD86 in non-retained fraction, which corresponds to M1 macrophages present in the COPD lung and the DC. Conversely, a down-expression of CD206 was detected in both retained and non-retained fractions following injection of M2 macrophages. This observation could most probably due to the secretion of IFN-γ following LPS exposition to the lung and which were found to down-regulate CD206 expression and enhance the expression of iNOS that, in turn, enhance nitric oxide synthesis [41].

Corroborating these findings, we also observed through immunohistochemistry that while F4/80 was observed as a universal marker for macrophages, there was an almost equal staining patterns in the expression of NOS2, a marker for M1 macrophages as well as Arginase1, a marker of M2 macrophages [42]. Prussian blue staining confirmed the presence of iron oxide nanoparticles and their co-localization with macrophages in tissue sections.

Although, this study provides preliminary evidence for the presence of a balanced number of M1 and M2 macrophages at this stage of COPD inflammation, which we believe continuously change their polarization state during the time course of inflammation and after injection of macrophages subpopulations, further confirmatory studies are needed to conclusively establish this phenomenon. It must be emphasized that a key component of the inflammatory response in COPD necessitates that pulmonary macrophages do not remain committed to a single activation profile [42]. They may regress to a resting state and subsequently be reactivated with a different polarization profile. It becomes increasingly evident that cross-talk of various signals at different levels of inflammation may help in the generation of particular macrophage profiles as needed by the host defense mechanisms.

## Conclusion

In conclusion, this study demonstrated for the first time the possibility of tracking and non-invasively monitoring intravenously injected bone marrow derived M1 and M2 macrophages subpopulations to the sites of inflammation in the lung of COPD model. Amine-modified PEGylated dextran-coated SPIO nanoparticles used in this study showed optimal biocompatibility and labeling efficiency, which allowed for better detection using non-invasive MRI. Investigation of macrophages polarization state one reaching the lung at 2 hours post injection demonstrated that during LPS-induced inflammation in COPD, a balanced proportion of macrophage subpopulations predominate even that a continuum switch in their polarization were occurring. Although, this study provides preliminary evidence of this process, the authors deem necessary further confirmatory studies to conclusively establish this phenomenon.

## Supporting Information

Figure S1 **Calibration curve of the different iron oxide concentrations.** Absorbance was measured at 351 nm.
(TIF)

File S1 **Supporting text.**
(DOCX)
