# Supplementary figures and images for: Preferential Macrophage Recruitment and Polarization in LPS-Induced Animal Model for COPD: Noninvasive Tracking Using MRI
Source: PLoS One. 2014 Mar 5;9(3):e90829. doi: 10.1371/journal.pone.0090829 (PMC3945006; doi:10.1371/journal.pone.0090829)

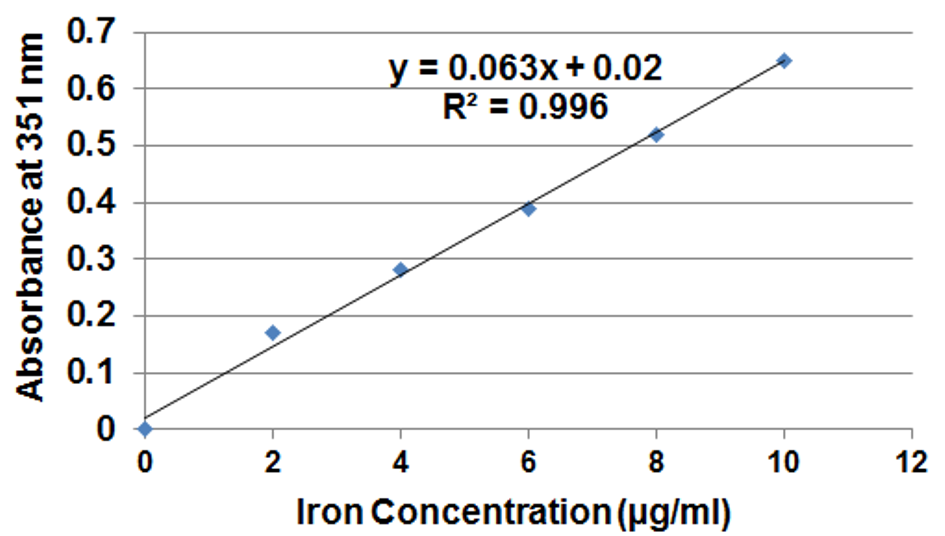

Supplement: Figure S1 — Calibration curve of the different iron oxide concentrations. Absorbance was measured at 351 nm. (TIF) [file pone.0090829.s001.tif]
