# Supplementary material for: Preferential Macrophage Recruitment and Polarization in LPS-Induced Animal Model for COPD: Noninvasive Tracking Using MRI
Source: PLoS One. 2014 Mar 5;9(3):e90829. doi: 10.1371/journal.pone.0090829 (PMC3945006; doi:10.1371/journal.pone.0090829)
Supplement: File S1 — Supporting text. (DOCX) [file pone.0090829.s002.docx]

**Nanoparticles relaxivities measurement:**

For the measurement of T1 relaxation time, an inversion-recovery fast imaging with steady state precession (IR-FISP) sequence was used with TR/TE = 4/2 ms, and an increasing inversion time starting from TI = 100 ms with 60 echoes.

For the measurement of T2 relaxation time, a Multi Spin Multi Echo (MSME) sequence was used with a TR of 2500 ms and increasing TEs starting from 12 ms with 15 echoes of 12 ms echo spacing.

For the measurement of T2* relaxation time, a Multi Gradient Echo (MGE) sequence was using with a TR of 1500 ms and increasing TEs starting from 4 ms with 15 echoes of 4 ms echo spacing.

T1, T2 and T2* relaxation times were automatically calculated using the Paravision image analysis software.

Relaxivities were then calculated as the slope of the linear regression generated from a plot of the measured relaxation rate (1/Ti, where i = 1, 2 or 2*) versus the concentration of the particles. (1/Ti) = (1/Ti(0)) + ri[SPIO] where Ti denotes the relaxation times of a suspension containing the particles and Ti(0) is the relaxation time of the solvent (water) without particles.

**Spectrophotometer calibration curve:**

In order to determine the iron content in labeled macrophages subsets, Ferrozine-based spectrophotometric assay was performed as previously described [[1](#_ENREF_1)]. In brief, tubes containing from zero to 10 μg of SPIO to generate a calibration line and 2x10^5^ cells were centrifuged at 3000 rpm for 5 min and the pellets were incubated at 110°C overnight with no cap on tubes. After evaporation of the liquid, 1 mL hydrochloric acid (5 M) was added, and the samples were further incubated, at 60°C for 4 h with cap on tubes, to prevent the acid evaporation. Then 0.5 mL solution from each tube was transferred to a separate cuvette, and 0.5 mL of 5 M hydrochloric acid was added to each cuvette after which the absorbance was measured at 351 nm, previously determined as the peak absorbance wavelength (data not shown). The iron concentration in labeled macrophages, expressed as pg of iron per cell, were then determined by comparing their absorbance to the calibration curve of the different iron oxide concentrations (Figure S1).

**References:**

1. Rad AM, Janic B, Iskander AS, Soltanian-Zadeh H, Arbab AS (2007) Measurement of quantity of iron in magnetically labeled cells: comparison among different UV/VIS spectrometric methods. Biotechniques 43: 627-628, 630, 632 passim.
